# A new potential cause of secondary hypertension

**DOI:** 10.3389/fcvm.2024.1458089

**Published:** 2024-08-29

**Authors:** Milos Mijalkovic, Dalila Sacic

**Affiliations:** ^1^ Polyclinic Kardio Medika, Nish, Serbia; ^2^ Clinic for Cardiology, University Clinical Center of Serbia, Belgrade, Serbia

**Keywords:** arterial hypertension, secondary hypertension, blood pressure, hyperinsulinemia, pancreatic insulinoma

## Introduction

Arterial hypertension is a well-known and leading cause of cardiovascular diseases and premature death worldwide. In the last few decades, the prevalence of arterial hypertension has been constantly increasing in low and middle-income countries of the world, as opposed to high-income countries (1).

According to the European guiedlines for the managment of hypertension from 2018 to 2023, arterial hypertension is defined when blood pressure is ≥140/90 mmHg, while the American College of Cardiology/American Heart Association (ACC/AHA) for the treatment of hypertension from 2017 redefine arterial hypertension as blood pressure values ≥130/80 mmHg. This change and redefinition of the ACC/AHA hypertension guidelines is based on the findings of numerous large prospective observational studies, as well as the results of randomized clinical trials, including the SPRINT study, which showed that intensive lowering of systolic blood pressure <120 mmHg, reduced the risk of cardiovascular death and all-cause mortality, to a greater extent than lowering systolic blood pressure ≤140 mmHg. When the new definition was applied to the general population of the United States of America, the prevalence of hypertension increased from 32% to 45.4%, while in the general population of China the increase in the prevalence of hypertension was even greater from 23.2% to 46.4% (1–4).

The global increase in the prevalence of primary arterial hypertension in the world, which does not have a clear etiology, requires greater attention and care, and a more detailed search for the possible causes of secondary hypertension. Secondary hypertension is significantly less common than primary arterial hypertension, its true prevalence is not precisely known, but it is assumed to be about 5%–15% of all hypertensive cases, and often remains unrecognized. Secondary forms of hypertension require specific diagnostic procedures, which allow to discover their specific causes, and to choose an effective drug treatment or an appropriate interventional treatment that controls or treats elevated blood pressure (3). Secondary hypertension can lead to target organ damage, independent of the effect of blood pressure itself, which can be remedied with appropriate treatment. Discovering the potential cause of secondary hypertension can lead to successful intervention, with the potential to improve quality of life, and reduce cardiovascular morbidity and mortality (5). Screening all hypertensive patients for secondary hypertension is not feasible or cost-effective, but there are some general clinical characteristics of patients that suggest they are more likely to have secondary hypertension and in whom screening should be considered after confirmation of elevated blood pressure by ambulatory blood pressure monitoring (4). Severe or resistant hypertension, the appearance of hypertension in persons under 30 years of age, malignant or accelerated hypertension, an acute rise in blood pressure from previously stable readings, a sudden increase in blood pressure in the elderly, should certainly arouse the suspicion of the existence of secondary hypertension (5). As is well known, the more common causes of secondary hypertension include obstructive sleep apnea, renal parenchymal disease, renovascular hypertension (atherosclerotic renovascular disease, fibromuscular dysplasia), primary hyperaldosteronism. Less common causes of secondary hypertension include pheochromocytoma, paraganglioma, acromegaly, Cushing's syndrome, hyperthyroidism, hypothyroidism, hyperparathyroidism, coarctation of the aorta, effects of alcohol and some drugs, chemotherapy agents, rare genetic causes of secondary hypertension, etc (3–5).

Pancreatic insulinomas are rare, mostly benign, small neuroendocrine tumors with an estimated incidence of 1–4 cases per million population annualy. They can be can be classified symptomatically into secreting and non-secreting (silent) tumors. The average age of patients is around 47 years, and they are more common in women than in men (1,4:1). Over the past few decades, the incidence of neuroendocrine tumors has been increasing, compared to the overall incidence of carcinomas (6).

Pancreatic insulinomas are characterized by chronic sustained hyperinsulinemia leading to recurrent hypoglycemia. There is evidence suggesting that hyperinsulinemia and insulin resistance may be the initial events in the genesis of secondary hypertension. The hypothesized relationship between hyperinsulinemia and hypertension occurs more frequently in white people than in black. There are several possible mechanisms of secondary hypertension caused by pancreatic insulinoma. Firstly, severe hypoglycemia due to insulinoma can trigger the release of catecholamines, leading to paroxysmal hypertension via activation of the sympathoadrenal system. Secondly, insulin can increase sodium retention in the kidneys, primarily in the distal nephron, as well as induce changes in the vascular system, contributing to an increase in blood pressure (6).

Only a few small studies have been done that are older and were published in the 90 s, which investigated the relationship between insulinoma of the pancreas and hypertension (7–9). The most significant is the long-term cohort study at the Mayo Clinic published in 1993, in which a solid cause-and-effect relationship between pancreatic insulinoma and significant hypertension was not proven, but the possibility of its existence was also not completely excluded (10).

However, it should be emphasized that the number of data and case reports showing a relationship between pancreatic insulinoma and significant hypertension is increasing.

Kaul et al. reported a 10-year-old girl with hypoglycemia with high insulin levels, distal symmetric motor-sensory axonal neuropathy, and hypertension, with normal urinary catecholamines (11). Ko Harada et al. described a case of a 65-year-old woman with pancreatic insulinoma, hypoglycemia and paroxysmal hypertension and elevated plasma and urinary catecholamines (12). Mijalkovic M. et al. presented a case of a 61-year-old man with pancreatic insulinoma, sleep apnea, hypoglycemia, and significant hypertension (6).

In conclusion, further research is needed to establish a definitive causal relationship between pancreatic insulinoma and secondary hypertension.

## References

[1] Mills KT Stefanescu A He J. The global epidemiology of hypertension. Nat Rev Nephrol. (2020) 16(4):223–37. 10.1038/s41581-019-0244-2 32024986 PMC7998524

[2] Vemu PL Yang E Ebinger J. 2023 ESH hypertension guideline update: bringing us closer together across the pond. JACC (2024) 83(5).

[3] Mancia G Kreutz R Brunström M Burnier M Grassi G Januszewicz A 2023 ESH guidelines for the management of arterial hypertension. J Hypertens. (2023) 41(12):1874–2071. 10.1097/HJH.0000000000003480 37345492

[4] Williams B Mancia G Spiering W Agabiti Rosei E Azizi M Burnier M 2018 ESC/ESH guidelines for the management of arterial hypertension. Eur Heart J. (2018) 39(33):3021–104. 10.1093/eurheartj/ehy339 30165516

[5] Sarathy H Salman LA Lee C Cohen JB. Evaluation and management of secondary hypertension. Med Clin North Am. (2022) 106(2):269–83. 10.1016/j.mcna.2021.11.004 35227430 PMC9728017

[6] Mijalkovic M Sacic D Pajovic S. A rare case of pancreatic insulinoma, sleep apnea, and hypertension. Cardiol Res Cardio Vasc Med. (2024) 9:243. 10.29011/2575-7083.100243

[7] Tsutsu N Nunoi K Kodama T Nomiyama R Iwase M Fujishima M. Lack of association between blood pressure and insulin in patients with insulinoma. J Hypertens. (1990) 8:479–82. 10.1097/00004872-199005000-00014 2163424

[8] Sawicki PT Heinemann L Starke A Berger M. Hyperinsulinaemia is not linked with blood pressure elevation in patients with insulinoma. Diabetologia. (1992) 35:649–52. 10.1007/BF00400257 1644243

[9] Fujita N Baba T Tomiyama T Kodama T Kako N. Hyperinsulinaemia and blood pressure in patients with insulinoma. Br Med J. (1992) 304:1157. 10.1136/bmj.304.6835.1157 1392796 PMC1882112

[10] O'Brien T Young WF Palumbo PJ O'Brien PC Service FJ. Hypertension and dyslipidemia in patients with insulinoma. Mayo Clin Proc. (1993) 68(2):141–6. 10.1016/S0025-6196(12)60161-X 8423694

[11] Kaul B Kaur P Tripathi M Khadgawat R Ammini AC Agarwala S An unusual cause of reversible axonal neuropathy and hypertension in a 10-year-old girl. J Clin Neurosci. (2012) 19(8):1196–7. 10.1016/j.jocn.2011.10.018 22613486

[12] Harada K Hanayama Y Hasegawa K Iwamuro M Hagiya H Yoshida R Paroxysmal hypertension induced by an insulinoma. Intern Med. (2017) 56(4):413–7. 10.2169/internalmedicine.56.7758 28202863 PMC5364194

